# Structural and Optical Properties of Graphene Quantum Dots−Polyvinyl Alcohol Composite Thin Film and Its Potential in Plasmonic Sensing of Carbaryl

**DOI:** 10.3390/nano12224105

**Published:** 2022-11-21

**Authors:** Nurul Illya Muhamad Fauzi, Yap Wing Fen, Faten Bashar Kamal Eddin, Wan Mohd Ebtisyam Mustaqim Mohd Daniyal

**Affiliations:** 1 Functional Nanotechnology Devices Laboratory, Institute of Nanoscience and Nanotechnology, Universiti Putra Malaysia, 43400 UPM Serdang, Selangor, Malaysia; 2 Department of Physics, Faculty of Science, Universiti Putra Malaysia, 43400 UPM Serdang, Selangor, Malaysia

**Keywords:** graphene quantum dots, polyvinyl alcohol, carbaryl, surface plasmon resonance

## Abstract

In this study, graphene quantum dots (GQDs) and polyvinyl alcohol (PVA) composite was prepared and then coated on the surface of gold thin film via the spin coating technique. Subsequently, Fourier transform infrared spectroscopy (FT-IR), atomic force microscopy (AFM), and ultraviolet-visible spectroscopy (UV–Vis) were adopted to understand the structure, surface morphology, and optical properties of the prepared samples. The FT-IR spectral analysis revealed important bands, such as O–H stretching, C=O stretching, C-H stretching, and O=C=O stretching vibrations. The surface roughness of the GQDs-PVA composite thin film was found to be increased after exposure to carbaryl. On the other hand, the optical absorbance of the GQDs-PVA thin film was obtained and further analysis was conducted, revealing a band gap *E_g_* value of 4.090 eV. The sensing potential of the thin film was analyzed using surface plasmon resonance (SPR) spectroscopy. The findings demonstrated that the developed sensor’s lowest detection limit for carbaryl was 0.001 ppb, which was lower than that previously reported, i.e., 0.007 ppb. Moreover, other sensing performance parameters, such as full width at half maximum, detection accuracy, and signal-to-noise ratio, were also investigated to evaluate the sensor’s efficiency.

## 1. Introduction

The last decade has witnessed an unprecedented number of studies investigating the synthesis and application of graphene-based materials, owing to their remarkable optical and mechanical properties [1,2,3]. Among the many members of the graphene family, graphene quantum dots (GQDs) are one of the most recent superstars of the carbon family. They have attracted significant attention because of their tunable band gap, which is caused by their smaller size than standard graphene sheets, excellent dispersibility, and biocompatibility [4,5,6]. Interestingly, GQDs can be compounded with solid materials and dispersed in common solvents [7,8]. To date, GQDs have demonstrated substantial potential in numerous applications, such as sensors [9], catalysis [10], energy devices [11,12], photothermal therapy [13,14], drug delivery [15], bioimaging [16,17], and solar cells [18]. Regarding optical sensing applications in the detection of pesticides, research indicates that synthesizing GQDs with a polymer can obtain highly sensitive material.

Based on literature analysis, several studies have detected pesticides using GQDs/magnetic silica beads-polypyrrole (PPy) [19], nitrogen-doped GQDs-polydopamine (PDA) [20], and sulfur-doped GQDs-polyvinyl alcohol (PVA) [21]. Among these materials, the PVA polymer has sparked considerable past and current interest, and studies are still being conducted to this day. PVA can also reportedly detect pesticides by synthesizing it with copper [22], encapsulated 5-aminofluorescein [23], cellulose silver nanoparticles [24], and gold nanoparticles [25,26]. It is worth mentioning that all of the reported materials have been used to detect different types of pesticides. PVA has been widely used amidst a range of conducting and non-conducting polymers, owing to its availability in different molecular weights and affordability [27]. Furthermore, PVA is non-toxic and exhibits notable film-forming features, strong water solubility, easy processability, and is biocompatible and biodegradable [28,29,30]. These remarkable properties make PVA an ideal candidate for many applications, including gas sensors [31], biomedical devices [32], drug delivery [33], fuel cells, and membrane technology [34].

According to previous research exploring GQDs and PVA-based materials for the optical detection of pesticides, the most commonly used optical methods include fluorescence, photoluminescence, colorimetry, and surface-enhanced Raman spectroscopy [35,36,37,38,39,40,41]. Although the results obtained have been satisfactory, these methods have some limitations, including the need for sophisticated equipment, complicated procedures for pre- analysis, and they are time consuming. The surface plasmon resonance (SPR) method is expected to eliminate these problems. SPR corresponds to the free electrons on the surface of a metallic nanoparticle after being excited by the photons of incident light with a certain angle of incidence, which are then propagated parallel to the metal surface [42,43,44,45,46,47,48,49]. SPR is also a highly effective approach for the optical and label-free detection of tiny molecules, possessing advantages, including simple and low-cost fabrication, noticeable sensitivity, and quick analysis times [50,51,52,53,54,55,56,57]. This approach is necessary to identify pesticides such as carbaryl (1-naphthyl N-methylcarbamate), which comprises one of the main agricultural pesticides used in the modern agricultural sector. This substance is dangerous for humans because it severely inhibits acetylcholine esterase and forms strong mutagenic N-nitrosocarbamates, which lead to nervous system problems [58,59]. Due to its toxicity and broad use, the detection of this environmental contaminant necessitates the development of sensitive analytical techniques to track levels of carbaryl and monitor bioaccumulation.

As the structural and optical properties of GQDs-PVA have not been extensively reported, this study attempted to characterize the structure, surface morphology, and optical properties of a GQDs-PVA nanocomposite using Fourier transform infrared spectroscopy (FT-IR), atomic force microscopy (AFM), and ultraviolet-visible spectroscopy (UV-Vis). Importantly, the fabrication of GQDs-PVA for carbaryl pesticide detection has yet to be investigated using SPR. Thus, this investigation will explore the potential sensing properties of GQDs-PVA for carbaryl. This study highlights that the future of GQDs research is boundless, particularly if upcoming studies focus on the ease of eco-friendly synthesis and improving GQDs-PVA materials.

## 2. Materials and Method

### 2.1. Material Preparation

**Figure 1:**
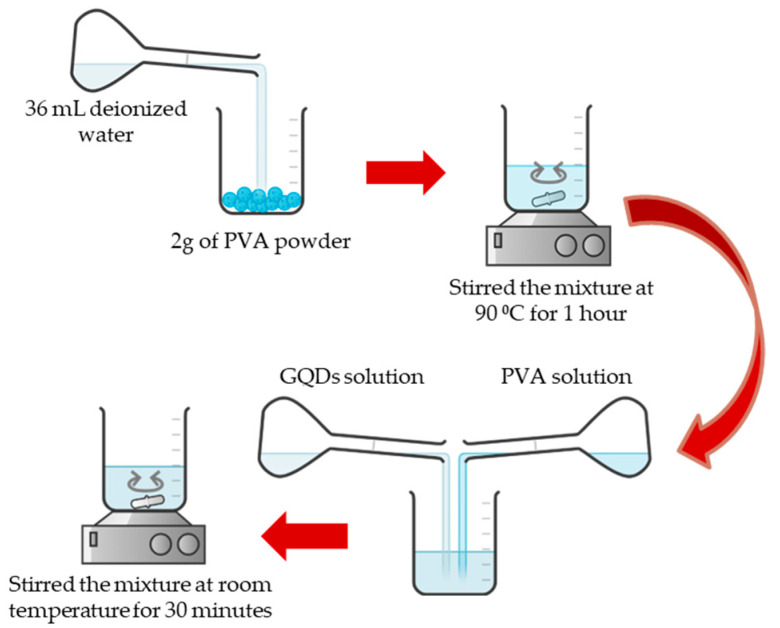
The schematic preparation of the composite solution.

PVA with an average molecular weight of 146,000–186,000 (87–89% hydrolyzed) and a stock solution (100 mg/mL) of carbaryl (98.0%) were obtained from Sigma-Aldrich (St. Louis, MO, USA). GQDs synthesized using the hydrothermal method (1 mg/mL) were provided by ACS Material (Pasadena, CA, USA). The solution emitted blue light (460 nm) when excited with a 365 nm UV beam. The purity of GQDs was more than 80% with a particle size of less than 5 nm. For preparation of the PVA solution, 2.0 g of PVA powder was mixed with 36 mL of deionized water. Then, the solution was stirred for 1 h with a magnetic stirrer at 90 °C. To generate the GQDs-PVA solution, the previously prepared PVA solution was mixed with the GQDs solution in a 1:1 volume ratio and stirred at room temperature for 30 min. Figure 1 shows the schematic preparation of the composite solution. Then, various concentrations of carbaryl solutions were prepared by diluting the appropriate amount in deionized water, using the diluting formula *M*_1_*V*_1_ = *M*_2_*V*_2_.

*M*_1_ = Stock concentration
*V*_1_ = Stock volume
*M*_2_ = Desired concentration
*V*_2_ = Desired volume

### 2.2. Sensing Layer Preparation

**Figure 2:**
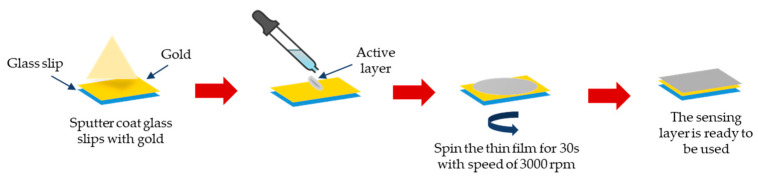
Steps involved in thin film preparation process.

First, the glass coverslip (24 × 24 × 0.1 mm^3^) was cleaned with acetone to eliminate any fingerprint marks or grime on the glass surface. The glass slip was coated with a 50 nm gold thin film using the SC7640 Sputter (Quorum Technologies, West Sussex, UK). The gold-coated glass slip was then deposited with a GQDs-PVA composite thin film using the spin coating method with the P-6708D Spin Coater (Inc. Medical Devices, Indianapolis, IN, USA). Approximately 0.50 mL of the sample solution was placed on the gold film and spun at 3000 rpm for 30 s. Figure 2 provides an overview of the steps involved in the thin film preparation process.

### 2.3. Characterization

The FTIR spectra of the GQDs, PVA, and GQDs-PVA solutions were recorded using an ALPHA II FTIR Spectrometer (Bruker, CA, USA) in ATR mode. Then, the surface morphology of the GQDs, PVA, and GQDs-PVA thin films were examined by using the Dimension Edge AFM (Bruker, CA, USA) in intermittent mode, since this mode allows high resolution for fragile and thin samples. A UV-3600 UV-VIS-NIR spectrophotometer (Shimadzu, Japan) was used to measure the absorbance and band gap of all of the thin films.

### 2.4. SPR Spectroscopy

**Figure 3:**
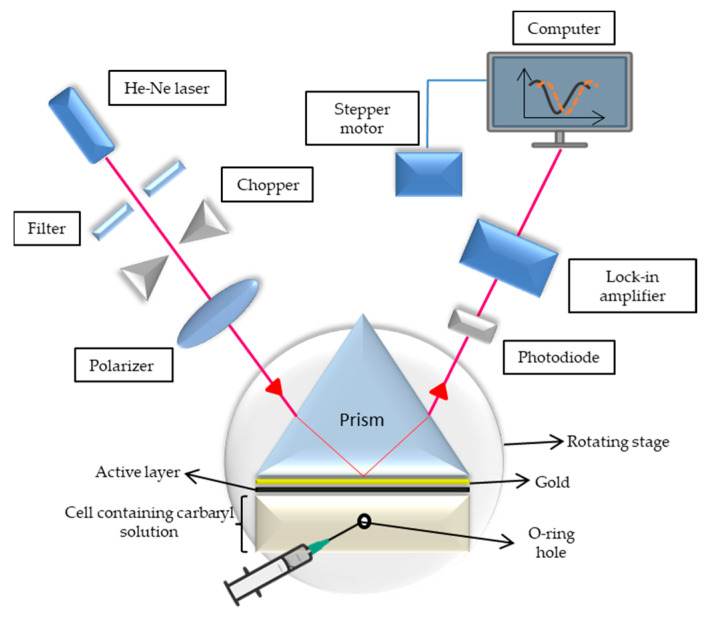
Schematic of surface plasmon resonance setup.

SPR occurs when a photon of incident light strikes a metal surface, usually a gold surface at specific conditions [60,61,62]. A component of the light energy couples with the metal surface layer’s electrons through the metal coating at a specific angle of incidence, causing the electrons to move as a response to excitation [63,64]. In an SPR sensor setup, incident light is utilized using a glass prism with a high reflecting index in the Kretschmann configuration of the attenuated total reflection (ATR) method [65]. The refractive index of the material closer to the metal surface determines the stated SPR angle at which resonance occurs, under the conditions of a constant light source wavelength and thin metal surface [66]. Thus, detection is accomplished by observing the change in the reflected light that is obtained on a detector. In this study, gold was used as the metal film because gold is inert, has high chemical stability, and efficiently absorbs light [67,68,69,70]. Then, GQDs-PVA was employed as an active layer to improve the sensitivity of the gold film for detecting pesticides. As a preliminary test, deionized water was injected into the cell, and the incidence angle for the reflectance curve was recorded as a reference. Subsequently, different carbaryl concentrations of 0.001, 0.008, 0.01, and 0.08 ppb were injected and left for 5 min to ensure that the carbaryl fully interacted with the GQDs-PVA layer. Figure 3 depicts the SPR spectroscopy sensor setup. The apparatus consisted of a laser, chopper, polarizer, sensor chip (gold layer and active layer), prism, photodiode, lock amplifier, stepper motor with resolution of 0.001°, and computer to display the changes in the reflected light.

## 3. Results and Discussion

### 3.1. Fourier Transform Infrared Spectroscopy

**Figure 4:**
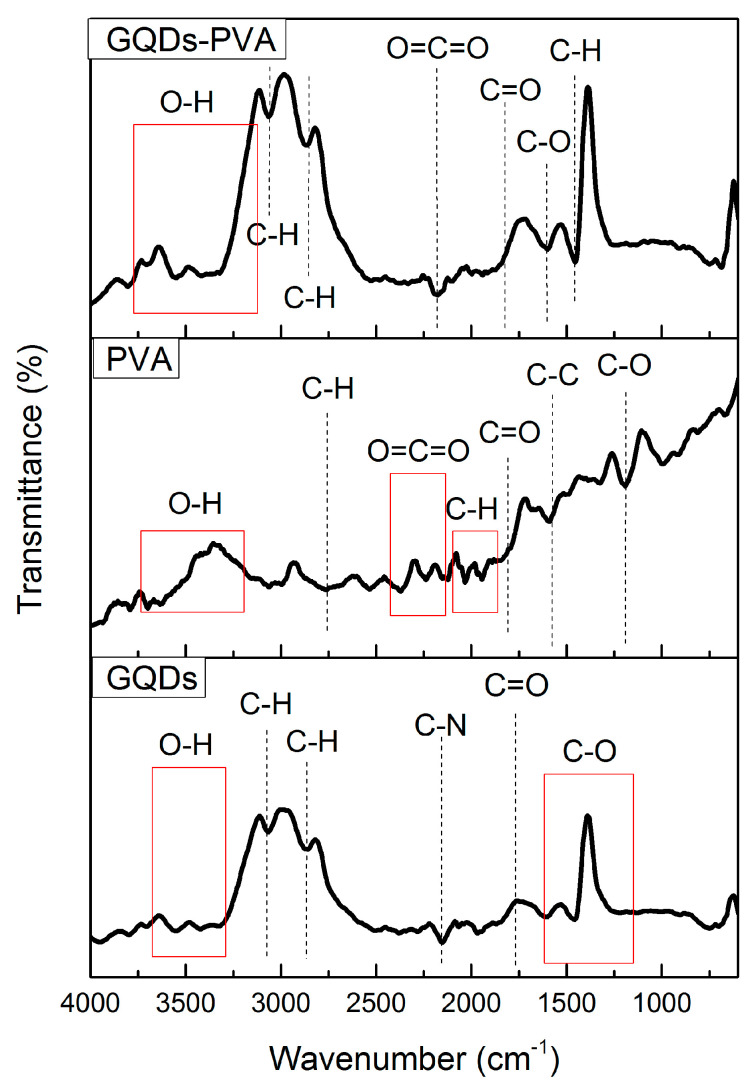
FTIR spectra of GQDs, PVA, and GQDs-PVA.

In this investigation, FTIR spectroscopy was used to identify the structural properties of GQDs, PVA, and GQDs-PVA films. For wavenumbers ranging from 4000 to 600 cm^−1^, the expected functional groups were revealed, consequently confirming the structures of all samples, as depicted in Figure 4. Referring to the FTIR spectrum of GQDs, the peak between 3314 and 3694 cm^−1^ was due to the characteristic O-H stretching vibration. The bands at 3075, 2867, 2145, 1767, and 1150–1616 cm^−1^ were assigned to C-H stretching (alkene), C-H stretching (alkane), which can indicate polycyclic aromatic hydrocarbons [71], C-N stretching, C=O stretching, and C-O stretching, respectively. Meanwhile, PVA exhibited the O-H stretching vibration, C-H stretching, O=C=O stretching, C-H bending of aromatic compounds, C=O stretching, C=C stretching, and C-O stretching, attributed to bands at 3196–3745, 2757, 2124, 1891–1987, 1726, 1575, and 1190 cm^−1^, respectively. These results were consistent with those reported by Yang et al. in 2018 [72]. After immobilization of GQDs with PVA composite, O-H stretching became intense at wavenumbers 3140–3786 cm^−1^. These results indicated that the reaction between GQDs functional groups and PVA groups occurred via interactions between the oxygenated groups in GQDs and the hydroxyl groups in PVA [73]. As can be seen in the FTIR spectrum of the GQDs-PVA film, C-H stretching (alkane), C-H stretching (alkene), O=C=O stretching, C=O stretching, C–O stretching, and C-H bending were attributed to bands at 2866, 3059, 2180, 1780, 1616, and 1465 cm^−1^, respectively. The integration of GQDs with PVA resulted in the formation of common characteristics and also a new peak, which appeared to be due to C-H bending.

### 3.2. Surface Morphology

**Figure 5:**
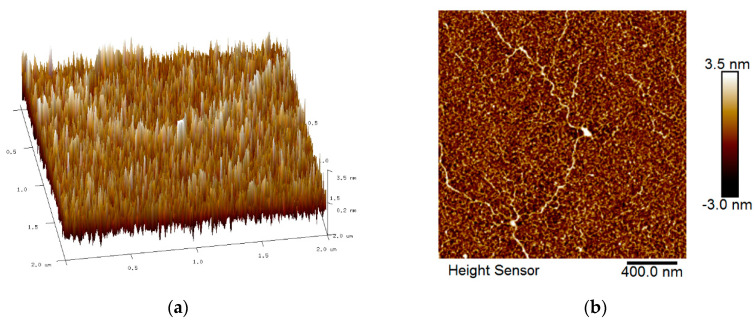
AFM images of GQDs thin film: (**a**) 3D image and (**b**) 2D image.

**Figure 6:**
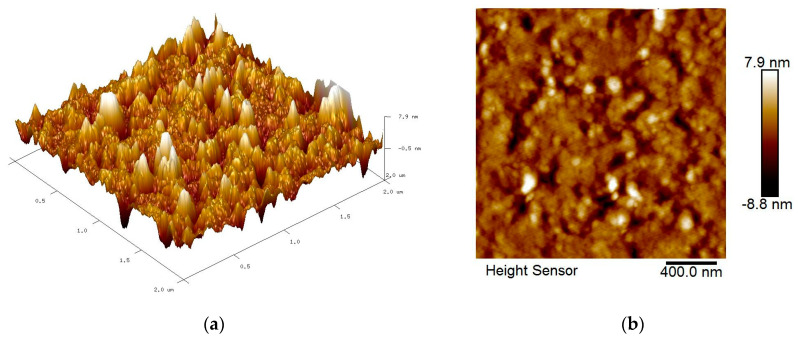
AFM images of PVA thin film: (**a**) 3D image and (**b**) 2D image.

**Figure 7:**
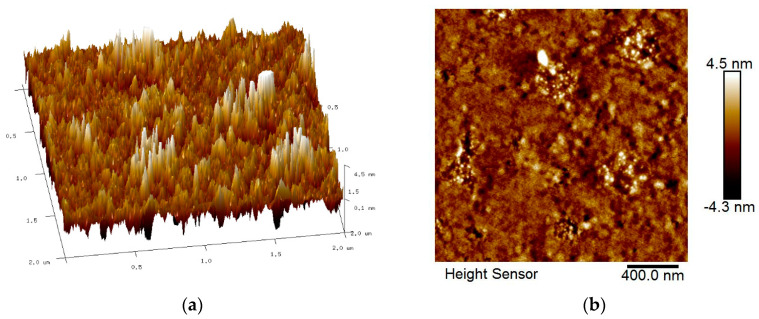
AFM images of GQDs-PVA thin film: (**a**) 3D image and (**b**) 2D image.

**Figure 8:**
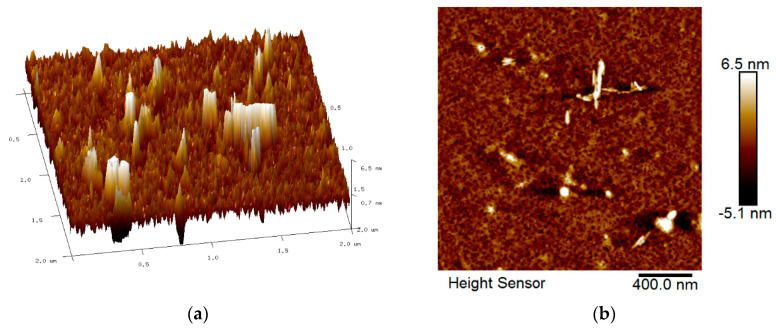
AFM images of GQDs-PVA thin film after contact with carbaryl: (**a**) 3D image and (**b**) 2D image.

The roughness features of all spin-coated thin films were identified by AFM with the scan size fixed to 2 µm × 2 µm. Figure 5, Figure 6, Figure 7 and Figure 8 show the AFM results for GQDs, PVA, GQDs-PVA, and GQDs-PVA after contact with carbaryl, following the composite materials being thoroughly coated on the surface of the gold thin film. As can be observed in Figure 5, the 2D image of the GQDs thin film revealed a relatively rough surface with a root mean square (RMS) roughness of 0.99 nm, similar to that observed by Anas et al. in 2019 [74]. Meanwhile, the 2D image of the PVA thin film in Figure 6 showed an even and circular surface structure, with an RMS roughness value of 2.86 nm. Then, the GQDs-PVA thin film in Figure 7 displayed an RMS roughness value of 1.12 nm with the surface appearance showing an even and granular structure. These changes in RMS roughness values may have been influenced by the formation of a network of GQDs nanoparticles with the PVA polymer [75]. After the GQDs-PVA thin film was exposed to carbaryl, the RMS roughness value increased to 1.38 nm, which proved the interaction of carbaryl with the GQDs-PVA thin film. The 2D image of the GQDs-PVA film after contact with carbaryl showed a fairly uniform surface structure with some white spots, which could be due to the carbaryl molecules, as depicted in Figure 8.

### 3.3. Optical Properties

**Figure 9:**
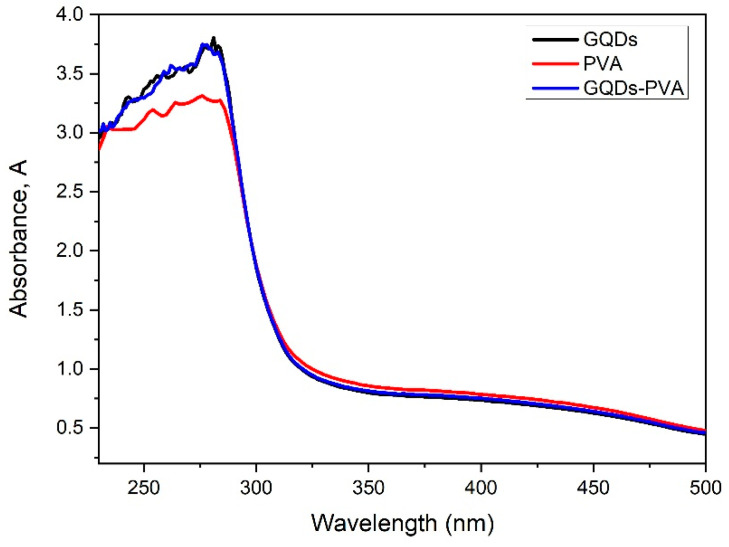
UV-Vis absorption spectra of GQDs, PVA, and GQDs-PVA thin films.

The optical properties of the composite materials were observed based on the UV-Vis spectrum with a spectral resolution of 0.1 nm. Thus, the absorption spectra of GQDs, PVA, and GQDs-PVA thin films were obtained at different wavelengths, ranging from 200 to 500 nm. As can be observed in Figure 9, the absorbance of GQDs showed the highest value of 3.8 at 281.7 nm. Meanwhile, the absorbance of PVA showed the lowest value of 3.3 at 275.4 nm. After GQDs were immobilized with the PVA layer, the absorbance value was approximately 3.7 at a wavelength of 276.3 nm. This peak may be attributed to the n–π* transition of the carbonyl group, as presented in the FTIR analysis [76].

The absorbance, *A*, is one of the important parameters for obtaining the optical band gap value for samples. Therefore, Beer Lambert’s law was used to interpret the data, as shown in the equation below [77]:

(1) $$a=2.303\frac{A}{t}$$

where *a* is the absorbance coefficient stated in units of m^−1^, and *t* represents the sample thickness in meters (m). Then, the Tauc equation was followed [78]:

(2) $$a=\frac{k{\left(hv-E_{g}\right)}^{\frac{1}{2}}}{hv}$$

where *k* indicates the constant value, *h* is Plank’s constant, *hv* represents the photon energy, and *E_g_* indicates the band gap. Then, Equation (2) was derived to obtain Equation (3) below:

(3) $${\left(ahv\right)}^{2}=k(hv-E_{g})$$

Plotting the graph of (*αhv*)^2^ against *hv* was estimated to obtain the band gap for the GQDs, PVA, and GQDs-PVA thin films, respectively. By extracting the extrapolated straight lines on the *x*-axis from the Tauc equation, he optical band gaps were obtained for all samples [79].

**Figure 10:**
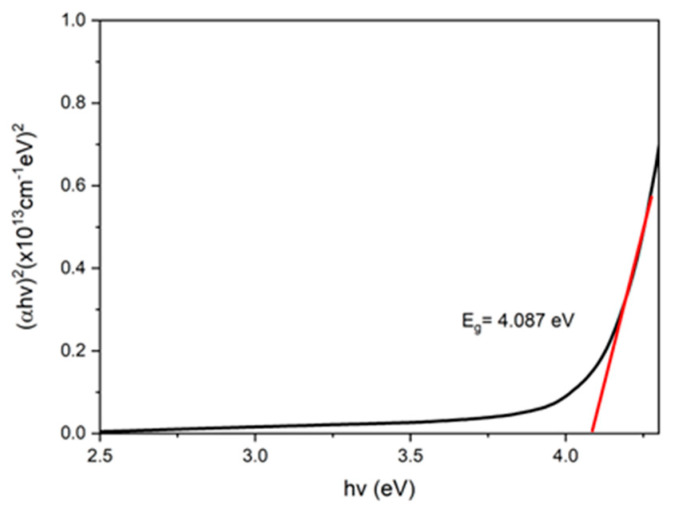
UV-Vis band gap energy of GQDs thin film.

**Figure 11:**
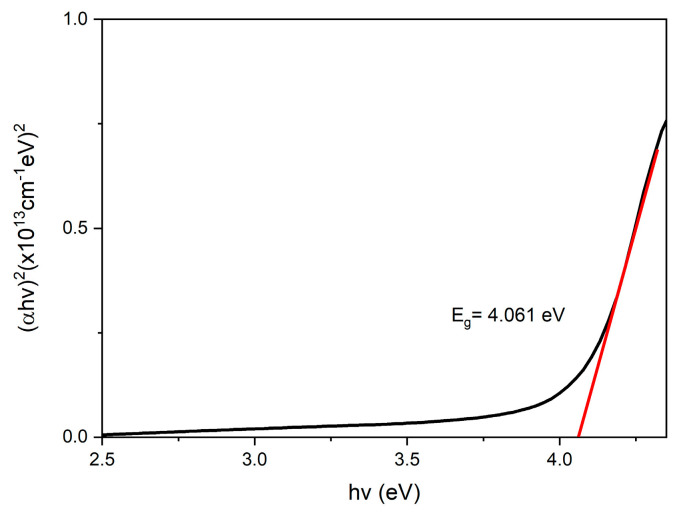
UV-Vis band gap energy of PVA thin film.

**Figure 12:**
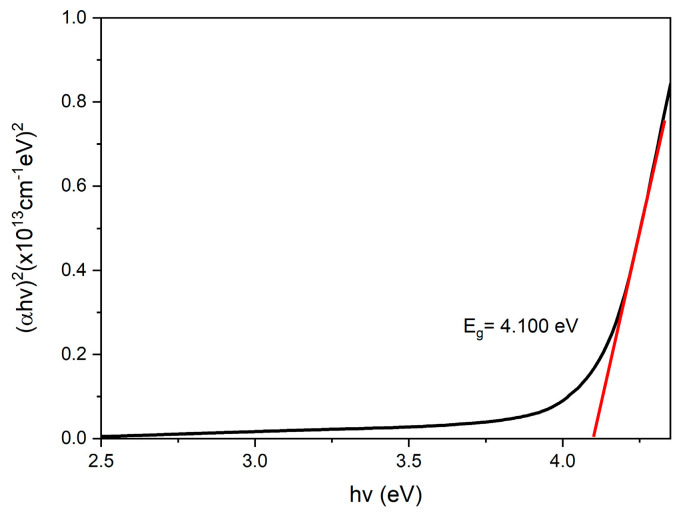
UV-Vis band gap energy of GQDs-PVA thin film.

Based on the graphs shown in Figure 10, Figure 11 and Figure 12, the optical band gap of GQDs was 4.087 eV, which aligned with the study by Bhatnagar et al. in 2017 [80]. The band gap for PVA was 4.061 eV and after GQDs were immobilized, the energy band gap was increased to 4.100 eV. The possible reason that the energy band gap of GQDs −PVA had the highest value compared to GQDs and PVA alone might be due to the quantum confinement effect. In the quantum confinement phenomenon, electrons and nanoscale holes in the semiconductor are trapped or confined [81]. As a result, the energy difference between full and empty states increases or widens the semiconductor’s band gap. According to Zhu et al. in 2017, the band gap in a quantum dot crystal is size dependent and can be altered to provide a range of energies between the valence and conduction bands. Additionally, because of quantum confinement, the band gap energy of a quantum dot increases as its size decreases [82].

### 3.4. Potential Plasmonic Sensing

**Figure 13:**
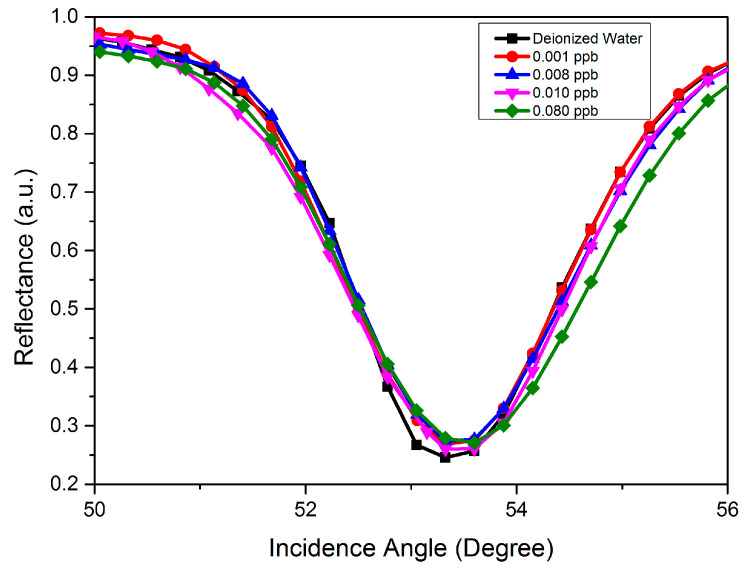
SPR reflectance curve as a function of incidence angle for gold thin film in the detection of different concentrations of carbaryl.

In the initial SPR analysis, the gold thin film was used as a baseline to compare the gold-modified GQDs-PVA layer. As a preliminary test, deionized water was injected into the cell and the incidence angle for the reflectance curve was recorded as a reference. Then, injections of carbaryl concentrations at 0.001, 0.008, 0.01, and 0.08 ppb were recorded. During the analysis, a sharp dip in the curve was obtained at a specific incidence angle that corresponded to the resonance angle, which resulted from the reflected light reduced because of the efficient transfer of energy to surface plasmons. As shown in Figure 13, the incidence angle for the reflectance curve of the gold thin film was recorded at 53.323°. As expected, the gold thin film had a weak binding capacity and was unable to distinguish changes in the refractive index of different concentrations of carbaryl, which led to approximately the same shift of the resonance angle.

**Figure 14:**
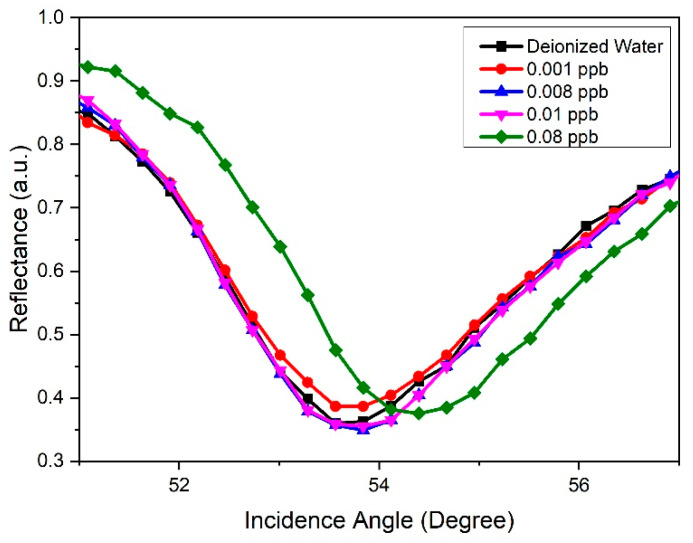
SPR reflectance curve as a function of incidence angle for GQDs-PVA thin film in the detection of different concentrations of carbaryl.

Then, the SPR analysis was continued by modifying the gold thin film with the GQDs-PVA solution. The SPR spectra for the GQDs-PVA sensor films in contact with deionized water and all concentrations of carbaryl are shown in Figure 14. The resonance angle obtained for deionized water was 53.694° while the resonance angle for carbaryl concentrations of 0.001, 0.008, 0.01, and 0.08 ppb were 53.709°, 53.787°, 53.809°, and 54.390°, respectively. The resonance angles increased and shifted slightly to the right with increasing carbaryl concentrations. The resonance angle incremental increasing trend can be attributed to the absorbed carbaryl filling the space between the GQDs-PVA layers. This finding was supported by previous research reporting that GQDs-PVA has a reticular structure to which small molecules can attach [75]. Moreover, electron changes on the surface of the GQDs-PVA also occurred in the presence of carbaryl adsorption, which led to changes in the refractive index when the carbaryl concentration increased. The electrons involved were probably from the COOH^−^ anion in GQDs and NH^3+^ cation in carbaryl. It also must be pointed out that this investigation had a limit of detection of 0.001 ppb, which was a lower limit detection than that reported by previous studies detecting carbaryl using various optical methods [36,37,38,39,40,41,83,84,85,86,87,88,89,90]. Comparisons with other studies are listed in Table 1.

To analyze the sensing parameters of GQDs-PVA thin film in the detection of carbaryl, the shift of resonance angle was introduced [91]. Based on the data in Table 2, the shift of the resonance angle was calculated by subtracting the resonance angle of different carbaryl concentrations from the resonance angle of deionized water. As a result, the shift of the resonance angle increased with increasing carbaryl concentration when in contact with GQDs-PVA thin film [92].

**Figure 15:**
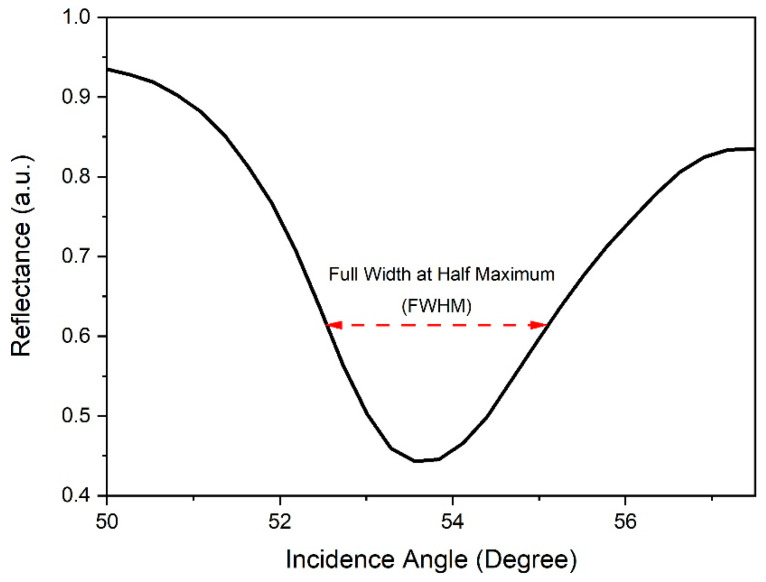
Illustration of FWHM, which represents half of the maximum value on the reflectance curve (for deionized water).

Full width at half maximum (FWHM) is a parameter that can indicate a sensor’s efficiency and accuracy [93]. The FWHM can be evaluated as the distance between the curve points at half the maximum peak level of the reflectance curve, as shown in Figure 15. Theoretically, a lower FWHM value indicates a shaper reflectance curve, which represents better sensor accuracy [94]. Through the FWHM data, detection accuracy (DA) can be obtained. DA is defined as the ratio of the shift of the resonance angle relative to the FWHM [95]. Therefore, DA is inversely proportional to the FWHM. In this analysis, the FWHM was experimentally measured from the reflectance curve for GQDs-PVA thin film after interaction with different concentrations of carbaryl.

**Figure 16:**
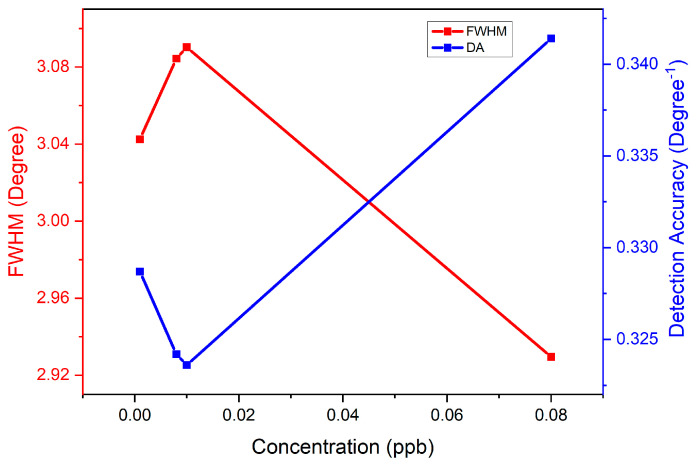
Graph of FWHM and DA versus concentration of carbaryl.

As shown in Figure 16, the FWHM was increased from 3.043° to 3.090° when the carbaryl concentration increased from 0.001 to 0.01 ppb. Then, from 0.01 to 0.08 ppb, the FWHM value decreased to 2.930°. Based on the data obtained, the trend in FWHM outcomes was not particularly stable. It fluctuates between increases and declines. As mentioned before, the FWHM value has an inverse relationship with DA, where a lower FWHM value gives better sensor detection accuracy. Thus, in this analysis, the detection of 0.08 ppb carbaryl was attributed to the smaller FWHM value of 2.930° and highest DA value of 0.341 degree^−1^.

Another important performance parameter of an SPR sensor is the signal–to–noise ratio (SNR). In other words, the SNR is evaluated to compare the level of the desired signal to the background noise [96]. In this SPR analysis, the value of the resonance angle shift was multiplied by DA to identify the SNR, as shown in Equation (4) below:

(4) $$SNR=\frac{\Delta \theta_{SPR}}{FWHM}=\Delta \theta_{SPR}\times DA$$

**Figure 17:**
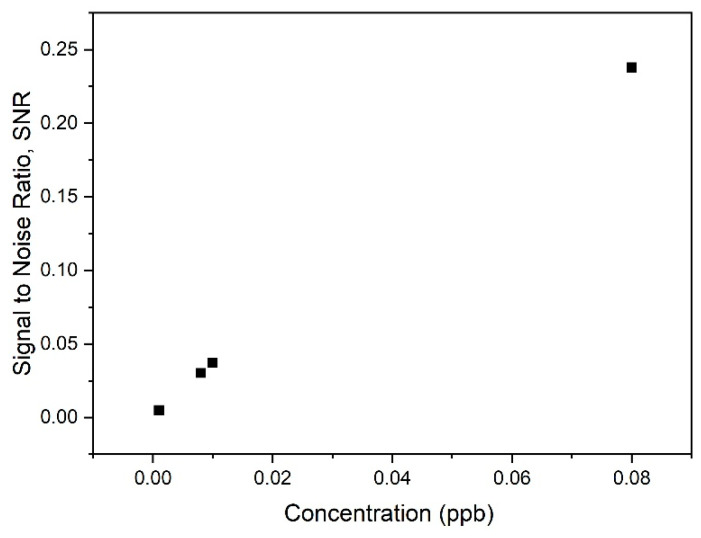
Graph of SNR versus carbaryl concentration.

From the data obtained for SNR values using Equation (4), the evolution of SNR with increasing carbaryl concentration was plotted. As illustrated in Figure 17, increasing the carbaryl concentration appeared to reduce the noise in the SPR signal, resulting in higher SNR values for the suggested sensor.

Table 3 summarizes the FWHM, DA, and SNR values for GQDs-PVA thin film in the detection of various concentrations of carbaryl. Based on the results presented, the best FWHM was obtained at the higher concentration of carbaryl (0.08 ppb) with a value of 2.930°. Notably, FWHM values are inversely proportional to DA values. Thus, in this analysis, 0.08 ppb carbaryl was detected at the highest DA value of 0.341 degree^−1^.

## 4. Conclusions

In this work, GQDs-PVA was successfully prepared using a simple chemical method and coated on gold film using the spin coating technique. Then, the structure, surface morphology, and optical and sensing properties were analyzed. FTIR analysis determined that the structure of the GQDs synthesized with PVA had the main components of O-H bonding, C=O bonding, C-H bonding, and O=C=O bonding. AFM analysis revealed that the surface morphology of the GQDs was affected by PVA with the appearance of a granular structure for GQDs-PVA. In addition, the value of RMS roughness increased after GQDs-PVA thin film was exposed to carbaryl solution and the surface became fairly uniform with white spots. Subsequently, the UV-Vis spectra of the thin films were evaluated to obtain the direct band gap of GQDs-PVA. This composite material had the highest energy band gap, 4.100 eV, compared to the single elements. Thus, the optical band gap data revealed that the GQDs-PVA exhibited semiconducting behavior. The GQDs-PVA thin film was investigated by SPR for potential sensing of carbaryl, where it showed high potential with a limit of detection of 0.001 ppb. Therefore, this study reveals the huge potential of GQDs-PVA for optical sensor applications in the detection of pesticides.

## Figures and Tables

**Table 1:** Comparison of this study with other carbaryl sensors using various materials in terms of limit of detection.

Method	Material	Limit of Detection (ppb)	Reference
Surface plasmon resonance	Monoclonal antibody	1.380	[36]
	Gold modified GQDs-PVA	0.001	This study
Colorimetry	Idophenyl acetate-acetylcholinesterase	2.010	[37]
	Gold nanoparticles	1.500	[38]
	Silver reduced-graphene oxide Carbon quantum dots-AuNPs-acetylcholinesterase	0.200	[39]
	p-acetamidobenzenesulfonyl azide–AuNPs	50.000	[83]
Fluorescence	Cadmium telluride quantum dots	0.120	[40]
	Graphene quantum dots	0.360	[88]
	3,5-di(2′,5′-dicarboxylphenyl)pyridine	6.700	[89]
	Flavourzyme-stabilized gold nanoclusters	0.470	[90]
Photoluminescence	Silicon quantum dots- acetylcholinesterase/choline oxidase	0.007	[84]
Chemiluminescence	Lum-AgNP	1000	[85]
Colorimetry and Chemiluminescence	Dual-graphitic carbon nitride/bismuth ferrite	0.033	[86]
High Fundamental Frequency Quartz Crystal Microbalance	Monoclonal antibody	0.050	[87]
Liquid Chromatography with tandem mass spectrometry	Acetylcholinesterase	20.000	[41]

**Table 2:** The resonance angle and shift of resonance angle for the detection of various concentrations of carbaryl (0−0.08 ppb).

Concentration of Carbaryl (ppb)	Resonance Angle, *θ* (Degree)	Shift of Resonance Angle, Δ*θ* (Degree)
0	53.694	0
0.001	53.709	0.015
0.008	53.787	0.093
0.01	53.809	0.115
0.08	54.390	0.697

**Table 3:** The FWHM, DA, and SNR values for GQDs-PVA thin film in the detection of carbaryl (0.001–0.08 ppb).

Concentration of Carbaryl (ppb)	Full Width Half Maximum (Degree)	Detection Accuracy (Degree^−1^)	Signal-to-Noise-Ratio
0.001	3.043	0.329	0.005
0.008	3.084	0.324	0.030
0.01	3.090	0.324	0.037
0.08	2.930	0.341	0.238

## Data Availability

Not applicable.
